# Educational and Resource Needs of Pelvic Health Physiotherapists: Context Matters

**DOI:** 10.1007/s00192-024-06036-3

**Published:** 2025-01-18

**Authors:** Corlia Brandt

**Affiliations:** University of the Witwatersrand, Johannesburg, South Africa

**Keywords:** Education, Needs, Pelvic health physiotherapy, Resources

## Abstract

**Introduction and Hypothesis:**

Evidence on health system challenges mostly relate to high-income countries. Lack of context-specific knowledge, educational opportunities, and access to resources among pelvic health care providers could be barriers to effective implementation of pelvic health services in South Africa. The aim of this study was to determine the patient and therapist profile, and the educational and resource needs of pelvic health physiotherapists in South Africa.

**Methods:**

Ninety-five pelvic health physiotherapists, recruited over 6 months, participated in a cross-sectional study during 2022–2023. Participants completed a REDCap survey covering the stipulated domains. Frequencies, percentages, and Chi-squared tests were used for data analysis.

**Results:**

The majority were employed in the private sector (*n* = 72; 75.8%) and had undergraduate training in pelvic health (*n* = 86; 89.5%); mostly in pre- and postnatal care (*n* = 69; 72.6%). Urinary incontinence was the most frequently seen condition (*n* = 81; 85.3%). Conditions were seen very seldom (*n* = 46; 48.4%) and most participants treated < 5 patients per month (*n* = 75; 78.9%), did not have patient educational material available (*n* = 58; 61.1%), preferred hard-copy formats (63.8%, *n* = 60), whereas 94.7% (*n* = 90) felt that there is a lack of patient resources. Participants (*n* = 66; 69.5%) indicated a need for educational opportunities for clinicians.

**Conclusions:**

This study highlights the contextualising of educational and resource needs in mid- to low-income countries, such as South Africa, which might be contrary to common beliefs and practices.

## Introduction

The health of women is currently flagged as one of the burdens of disease globally, although poverty, gender inequity, poor socio-economic status, sexual and gender-based violence (e.g. female genital mutilation), ageing populations and increased survival rates raise major concerns regarding the morbidity of women on the African continent [1].

Pelvic floor dysfunction may be associated with uterine or cervical cancer and treatment, HIV, ante- and post-natal complications, pulmonary disease, non-communicable disease, sexual trauma, pelvic fractures, spinal cord injury or other conditions that have a concerning prevalence on the African continent. This is reflected in the high reported prevalence rates of up to 60% of pelvic floor dysfunction, estimated in some low- to mid-income countries [2]. A prevalence of stress urinary incontinence of 52% has also been reported in Sub-Saharan Africa, although this may be an under-estimation owing to under-reporting and pelvic floor dysfunction still being an undertreated and under-investigated condition [3].

These concerns run parallel to the fact that little attention has been given at undergraduate and postgraduate level globally to Pelvic and Women`s Health education and content delivery [4], increasing the gap in service delivery and research we are currently experiencing in this field on a global scale [5, 6]. The field of pelvic health in physiotherapy has only emerged extensively in South Africa during the past decade and is a developing field in physiotherapy when compared with other areas. In some other African countries, such as Zambia and Botswana, this field and its development is still only in its infancy.

Although there is a growing body of evidence that outlines the effectiveness of conservative treatment in the management of pelvic floor dysfunction in different populations, most of the evidence on which clinical decisions regarding the management of pelvic floor dysfunction are made are based on evidence from mid- to high-income countries [7]. Several contextual factors relevant to the African continent may, however, lead to ineffective implementation of these management strategies. These factors include a lack of trained clinicians in the field, a lack of resources (human and financial), a lack of referral systems, a lack of patient education, poor accessibility to pelvic and women’s health services and poor support to newly qualified clinicians (Brandt, 2022, IUGA congress presentation). The factors may overcomplicate the task of the already isolated and perhaps overburdened clinician in a public or rural area, who received minimal undergraduate training in this field, leading to difficulties in providing quality care to the patients under challenging and unique conditions.

Although studies have looked at competency frameworks in pelvic health [4], topics such as perspectives pertaining to pelvic health physiotherapy [8], the nature and extent to which pelvic health content is incorporated in entry-to-practice physiotherapy programs (in Canada) [9], and suggested the need to increase training and qualified clinicians at an advanced level of pelvic health [5], none has looked into the contextual needs, training and educational support of the qualified physiotherapists in under-serviced and challenging health systems, such as on the African continent.

The aim of the study was therefore to determine the patient and therapist profile, and the educational and resource needs of pelvic health physiotherapists in South Africa.

## Materials and Methods

The cross-sectional study was approved by the relevant institution’s ethics committee. A convenience sampling method was used, including any physiotherapist who qualified with a Bachelor’s degree at an accredited higher education institution, and currently living and working in South Africa as a physiotherapist. Physiotherapists with no interest in, or not practicing in pelvic and women’s health, were excluded from the study.

There are approximately 6,000 qualified physiotherapists in the country, 250 (4%) of whom have a special interest in pelvic and women’s health physiotherapy. These physiotherapists work in a private (80%), public or rural setting (20%), spread throughout the nine provinces in South Africa. Patients visiting the clinics in the private sector usually have a higher socio-economic status and/or medical insurance to help cover their costs. The opposite applies to the patient population served by the public and rural sectors.

Based on the population size, a proportion of 4%, power of 80%, a 5% margin of error, a sample size of 58 were calculated. Adjusting for a fall-out rate of 20%, a total sample size of 70 was estimated.

The electronic survey, using REDCap (a secure web platform for building and managing online databases), was distributed via different organisational networks, health networks and social media platforms over a period of 6 months. The online questionnaire was preceded by an information document outlining the ethical concepts and purpose of the study. Subsequent completion of the questionnaire by the participant implied informed consent. The questionnaire included 13 questions covering different domains such as demographic information, educational history and needs, patient profile, and resource needs related to the pelvic health services that they render. The questionnaire was piloted on five participants before commencement of the main study. The pilot study followed the same procedures as the main study, and as no changes were made following the pilot study, the data from the pilot were included in the final analysis.

Data analysis was done using STATA Version 18.0 to summarise the frequencies and percentages for categorical data and means and standard deviations for the continuous data with a normal distribution. The Chi-squared test was used to determine relevant associations (α = 0.05).

## Results

Table 1 summarises the demographic information, educational history and needs of the physiotherapists, patient profile encountered by them, and their resource needs related to improving pelvic health services.

**Table 1:** Summary statistics regarding demographic information, educational history, needs of pelvic health physiotherapists, and patient profile encountered (*n* = 95)

Survey questions	Percentage (*n*)
In which health care setting are you currently practicing or employed?	
Private	75.8 (72)
Public	21.1 (20)
Rural	1.1 (1)
Higher education	0
Other	2.1 (2)
What is your highest qualification in pelvic and women's health physiotherapy?	
BA or BSc in Physiotherapy	89.5 (85)
Postgraduate Diploma	7.4 (7)
Master's degree	7.4 (7)
Doctoral degree	0
Other	10.5 (10)
Which topics did your undergraduate training in pelvic and women's health include?	
Decreased tone	47.7 (45)
Increased tone	18.9 (18)
Ante- and postnatal	72.6 (69)
Anorectal	4.2 (4)
Menopause	15.8 (15)
Adolescence	3.2 (3)
Men's health	4.2 (4)
Sexual health	8.4 (8)
Pelvic pain	26.3 (25)
Assessment of pelvic floor dysfunction	12.6 (12)
Pelvic organ prolapse	15.8 (15)
Gynaecological cancer	11.6 (11)
Other	10.5 (10)
How often do you treat patients with pelvic floor dysfunction?	
Every day	10.5 (10)
Every week	11.6 (11)
Every month	4.2 (4)
Every 3 months	13.7 (13)
Very seldom	48.4 (46)
Never	11.6 (11)
How many patients with pelvic floor dysfunction do you treat in a month?	
<5	78.9 (75)
5–10	6.3 (6)
11–20	9.5 (9)
>20	5.3 (5)
What is the most common pelvic floor dysfunction you encounter in your area?	
Pelvic organ prolapse	33.7 (32)
Urinary incontinence in women	85.3 (81)
Urinary incontinence in men	10.5 (10)
Urinary incontinence in children	3.2 (3)
Constipation	27.4 (26)
Sexual pain or dysfunction	21.1 (20)
Pelvic pain	45.3 (43)
Ante- and postnatal related	38.9 (37)
Faecal incontinence	2.1 (2)
Menopause associated	21.1 (20)
Erectile dysfunction	3.2 (3)
Gynaecological cancer	6.3 (6)
In which of the following populations do you routinely screen for pelvic floor dysfunction?	
Referred for pelvic floor dysfunction	52.6 (50)
Sport injuries	10.5 (10)
Neuromusculoskeletal disorders	58.9 (56)
Neurological	15.8 (15)
Patients in the intensive care units	4.2 (4)
Pelvic fractures	25.3 (24)
Athletes	9.5 (9)
Ante- and postnatal	53.7 (51)
None	13.7 (13)
Do you inform or educate vulnerable populations on pelvic health?	
Yes	61.1 (58)
No	38.9 (37)
Do you have educational material available that you can provide to patients on pelvic health?	
Yes	38.9 (37)
No	61.1 (58)
Do you think there are enough reliable resources available for patients on pelvic health?	
Yes	5.3 (5)
No	94.7 (90)
Which format or mode of information would most of your patients prefer to have access to?	
Short Message Services (SMS)	4.3 (4)
WhatsApp	41.5 (39)
Internet/websites	42.6 (40)
Videos	34 (32)
Photos	27.7 (26)
Hard copies	63.8 (60)
Do you think there is enough educational opportunities available for physiotherapists to improve their knowledge and skills on pelvic and women`s health management?	
Yes	30.5 (29)
No	69.5 (66)
Which training opportunities or resources on pelvic and women`s health would you be most interested in?	
Online workshops or courses	
Face-to-face sessions	
Clinical experience	
Website with resources	

A total of 95 responses were received, and a power of up to 99% was calculated based on a post-hoc power calculation for cross-sectional studies (using *n* = 72 as the exposed group, *n* = 20 as the non-exposed group). Prevalence differences ranged between 48–63% for some factors. Most of the sample were working in the private sector 75.8% (*n* = 72), whereas none of the respondents was working in a higher education setting. Ninety percent (*n* = 85) of the sample only had undergraduate training in pelvic and women’s health, whereas none of the respondents had a qualification on a PhD level. The topic mostly covered at undergraduate level on pelvic and women’s health was ante- and postnatal physiotherapy 72.6% (*n* = 69).

Although practising with a special interest in pelvic and women’s health, only 10.5% (*n* = 10) of the respondents saw patients with pelvic floor dysfunction daily. Seventy-nine percent (*n* = 75) saw fewer than five patients with pelvic floor dysfunction per month. The most frequently reported condition that respondents encountered was female urinary incontinence (85.3%, *n* = 81), whereas pelvic pain was the second most frequently reported condition (45.3%, *n* = 43). Patient populations mostly screened for pelvic floor dysfunction included those presenting with neuromusculoskeletal disorders (58.9%, *n* = 56), patients referred for conservative management of pelvic floor dysfunction (52.6%, *n* = 50), and ante- and postnatal patients (53.7%, *n* = 51). The majority reported that they inform or educate vulnerable populations on pelvic health (61.1%, *n* = 58), but a similar percentage of participants (61.1%, *n* = 58) reported that they do not have enough educational material available to share with the patients. Ninety-five percent (*n* = 90) were of the opinion that there are not enough reliable resources available for patients to access. Patients still seemed to prefer hard copies when it comes to modes of sharing information (63.8%, *n* = 60).

Participants reported a need for increased educational opportunities to improve their knowledge and skills (69.5%, *n* = 66), with online workshops or courses as the most preferred activity format (70.5%, *n* = 67) and clinical experience the least preferred format that they would like to engage in (38.9%, *n* = 37).

Considering the contextual differences between the public and private working environments, data were further analysed for possible associations (Table 2).

**Table 2:** Associations between survey variables and the health care sector (*n* = 95)

Variable	Private sector (*N* = 72), *n* (%)	Public sector (*N* = 20), *n*	Total, *n*	Chi-squared	*p* value
What is your highest qualification in pelvic and women's health physiotherapy?				4.58	0.205
BA or BSc in Physiotherapy	55 (74.32)	19 (25.68)	74 (100.00)		
Postgraduate Diploma	6 (100.00)	0	6 (100.00)		
Master's degree	7 (100.00)	0	7 (100.00)		
Doctoral degree	0	0	0		
How often do you treat patients with pelvic floor dysfunction?				5.24	0.387
Every day	9 (90.00)	1 (10.00)	10 (100.00)		
Every week	10 (90.91)	1 (9.09)	11 (100.00)		
Every month	2 (66.67)	1 (33.3)	3 (100.00)		
Every 3 months	9 (81.82)	2 (18.18)	11 (100.00)		
Very seldom	32 (69.57)	14 (30.43)	46 (100.00)		
Never	10 (90.91)	1 (9.09)	11 (100.00)		
How many patients with pelvic floor dysfunction do you treat in a month?				3.51	0.320
<5	54 (75.00)	18 (25.00)	72 (100.00)		
5–10	6 (100.00)	0	6 (100.00)		
11–20	7 (77.78)	2 (22.2)	9 (100.00)		
>20	5 (100.00)	0	5 (100.00)		
What is the most common pelvic floor dysfunction you encounter in your area?					
Urinary incontinence in women				4.58	0.032*
Yes	58 (74.36)	20 (25.64)	78 (100.00)		
No	14 (100.00)0	0	14 (100.00)		
Pelvic pain				0.95	0.331
Yes	34 (82.93)	7 (17.07)	41 (100.00)		
No	38 (74.51)	13 (25.49)	51 (100.00)		
Ante- and postnatal related				0.1	0.751
Yes	28 (80.00)	7 (20.00)	35 (100.00)		
No	44 (77.19)	13 (22.81)	57 (100.00)		
In which of the following populations do you routinely screen for pelvic floor dysfunction?					
Referred for pelvic floor dysfunction				5.34	0.021*
Yes	33 (68.75)	15 (31.25)	48 (100.00)		
No	39 (88.64)	5 (11.36)	44 (100.00)		
Neuromusculoskeletal disorders				2.7	0.100
Yes	47 (83.93)	9 (16.07)	56 (100.00)		
No	25 (69.44)	11 (30.56)	36 (100.00)		
Ante- and postnatal				0.05	0.826
Yes	38 (79.17)	10 (20.83)	48 (100.00)		
No	34 (77.27)	10 (22.73)	44 (100.00)		
Do you inform or educate vulnerable populations on pelvic health?				1.02	0.313
Yes	45 (81.82)	10 (18.18)	55 (100.00)		
No	27 (72.97)	10 (27.03)	37 (100.00)		
Do you have educational material available that you can provide to patients on pelvic health?				0.183	0.669
Yes	29 (80.56)	7 (19.44)	36 (100.00)		
No	43 (76.79)	13 (23.12)	56 (100.00)		
Do you think there is enough reliable resources available for patients on pelvic health?				1.469	0.226
Yes	5 (100.00)	0	5 (100.00)		
No	67 (77.01)	20 (22.99)	87 (100.00)		
Which format or mode of information would most of your patients prefer to have access to?					
WhatsApp				1.61	0.205
Yes	33 (84.62)	6 (15.38)	39 (100.00)		
No	39 (73.58)	14 (26.42)	53 (100.00)		
Internet/websites				10.98	0.001*
Yes	37 (94.87)	2 (5.13)	39 (100.00)		
No	35 (66.04)	18 (33.96)	53 (100.00)		
Hard copies				5.76	0.016*
Yes	40 (70.18)	17 (29.82)	57 (100.00)		
No	32 (91.43)	3 (8.57)	35 (100.00)		
Do you think there is enough educational opportunities available for physiotherapists to improve their knowledge and skills on pelvic and women`s health management?				8.33	0.004*
Yes	28 (96.55)	1 (3.45)	29 (100.00)		
No	44 (69.84)	19 (30.16)	63 (100.00)		
Which training opportunities or resources on pelvic and women`s health would you be most interested in?					
Online workshops or courses				0.01	0.942
Yes	51 (78.46)	14 (21.54)	65 (100.00)		
No	21 (77.78)	6 (22.22)	27 (100.00)		
Face-to-face sessions				2.8	0.094
Yes	39 (72.22)	15 (27.78)	54 (100.00)		
No	33 (86.84)	5 (13.16)	38 (100.00)		
Clinical experience				0.37	0.543
Yes	27 (75.00)	9 (25.00)	36 (100.00)		
No	45 (80.36)	11 (19.04)	56 (100.00)		
Website with resources				0.85	0.356
Yes	51 (80.95)	12 (19.05)	63 (100.00)		
No	21 (72.41)	8 (27.59)	29 (100.00)		

Table 2 shows that there was an association between the probabilities of being in the public sector on condition that women with urinary incontinence are commonly seen, compared with the probability of women with urinary incontinence not being the most common condition encountered in the public sector (*X*^2^(1) = 4.58, *p* = 0.032).

Associations were also found regarding the variables related to the public sector and probabilities of screening women who were referred for pelvic floor dysfunction (*X*^2^(1) = 5.34, *p* = 0.021), the internet or websites not being the preferred choice of format to share patient information (*X*^2^(1) = 10.98, *p* = 0.001), the use of hard copies as the preferred format of sharing patient information (*X*^2^(1) = 5.76, *p* = 0.016), and the opinion that there are not enough educational opportunities available for physiotherapists (*X*^2^(1) = 8.33, *p* = 0.004).

## Discussion

The findings from the survey highlighted several areas of concern. The South African health care system is a two-tier system, divided into two sectors, namely, public and private, which differs vastly with regard to socio-economic factors, resource availability and access to the services. The population served by the public sector is of lower socio-economic status and constitutes approximately 84% of the South African population [10]. Interestingly, the findings from our survey indicated a disproportionate distribution of pelvic health physiotherapists in the health care systems, as 75.85% of the respondents were working in the private sector (serving a higher income population). As the primary (public) health care systems are the gatekeepers to secondary health care [10], it implies that many patients with pelvic floor dysfunction might not get picked up by the system, and therefore not receive the necessary interventions or referral, either in the primary care setting or at secondary (specialised) level. This may further contribute to underreporting of pelvic floor dysfunction in these and other low- and mid-income settings when epidemiological studies are carried out [3].

Considering the latter statement, majority of the respondents in the survey (85.3%) indicated that urinary incontinence in women is the most common condition encountered. This aligns with global epidemiological studies indicating a high prevalence of urinary incontinence, ranging between 25 and 45% in adult women [11]. However, these conclusions are based on findings coming mainly from high-income countries, with very little literature available from African countries. A recent sub-analysis of a systematic review based on studies carried out in Sub-Saharan Africa, indicated prevalences of approximately 52% for stress urinary incontinence, 21% for urge urinary incontinence and 27% for mixed urinary incontinence [3].

When looking at the most common conditions encountered namely, urinary incontinence, pelvic pain and ante- and post-natal conditions, one should consider whether this could be due to the fact that participants were mainly trained in these areas at undergraduate level, equipping them to manage these patients; and/or whether it was related to the fact that these conditions also coincided with the populations that were mainly screened by them. Further analysis did, however, not reveal any associations between these variables (*p* > 0.05).

The concern is, though, that despite a high prevalence of reported pelvic floor dysfunction, 46% of the sample reported that they treat pelvic floor dysfunction very seldom. It related to the finding that 79% of the sample saw less than five patients with pelvic floor dysfunction per month. These findings were not associated with working in a specific health care setting, and it could be speculated that it is perhaps related to poor referral, accessibility or perhaps limited financial resources and patients covered by medical insurance. A cross-sectional study conducted at McMaster University amongst different health care professional students indicated a lack of knowledge regarding scope of practice, activities and application of pelvic health physiotherapy, which could in turn lead to poor referral practices [8].

The latter findings also highlight the importance of advocacy and patient education regarding pelvic floor dysfunction. Although 61.1% of the sample indicated that they inform or educate vulnerable populations on pelvic health, a similar percentage indicated that they do not have enough material available to share with patients. An overwhelming majority of 95% also indicated that there are not enough reliable resources for patients to access. This might be surprising as increasing efforts are constantly made by several organisations and institutions to improve resources and materials to be used in clinical settings, illustrated by multiple online resources in this field [12].

The accessibility and reach that technology and artificial intelligence are introducing has led to a move towards these platforms and formats as preferred methods of sharing information or resources, in an effort to revolutionise health care [13]. However, in mid- and low-income countries, contextual factors need to be considered when implementing intervention strategies. This was emphasised by the findings that 63.8% of the sample preferred to share information with patients using hard copy formats, whereas there was also a clear association with the type of sector (public) and preference of these more simplified formats of patient material. Effective patient education and formats is an essential factor to consider, as poor education may lead to miscommunication and misunderstanding on the patient's behalf, lack of trust and informed decision-making, and ultimately poor health [14].

Contrary to the latter finding, when participants had to indicate their preferred format of training for themselves, online options were mostly indicated. The contextual factor to consider here is the fact that it might be time- and cost-effective for the clinicians to attend this format of training, compared with in-person meetings, which would be more time-consuming and costly. The concern is, however, that practical training and clinical experience or mentoring requires an in-person format and is an important aspect of competency of pelvic health clinicians [15]. This hurdle would need to be addressed when promoting post-graduate training of which clinical competency forms a large component. As indicated in the survey, there is an urgent need to improve the postgraduate qualifications of pelvic health physiotherapists, which may improve the quality and quantity of health care, services and reach related to patients with pelvic floor dysfunction, specifically in the underserved public and rural areas. This goal is reiterated by organisations such as World Physiotherapy, urological and urogynaecology associations and societies, encouraging educators to strive for training and excellence in the profession and improved patient care [15]. Challenges, such as lack of educators in this field in higher education institutions, as identified in our study, will, however, need to be addressed at the same time.

Although this study was carried out in South Africa, the context may be similar to other low- or mid-income countries, or even some high-income countries when considering specific geographical or resource settings. A scoping review by Van Zyl et al. [16] identified nine themes that could be related to defining low-resource settings, namely influence of beliefs and practices; human resource limitations; geographical and environmental factors; restricted social resources; research considerations and challenges; paucity of knowledge; underdeveloped infrastructure; suboptimal service delivery in health care; and financial pressure [16]. The baseline data should therefore be used to further investigate implementation strategies to improve pelvic health care in populations and decrease the burden of pelvic floor dysfunction in mid- and low-income countries or resource settings. More detailed surveys and longitudinal studies can elaborate on contextual factors (association and risk) such as culture, beliefs, educational level of patients, language barriers, amongst other factors, and postgraduate education, to help to address the generic issues identified in this study. Implementation studies to, for example, investigate context-specific online training opportunities and resources, should be undertaken, evaluated and scaled up to achieve the desired outcomes and health needs in mid- and low-income countries or resource settings. The information can guide clinicians to improve their skills, services and patient care by addressing context-specific needs.

## Conclusion

This study is unique as it investigated the educational and resource needs of physiotherapists with a special interest in pelvic health, in a mid-income country/resource setting. The study highlighted findings that are contrary to common beliefs, such as the lack of pelvic health physiotherapists working in higher education institutions and in the public and rural sectors; the lack of formal postgraduate qualifications in this field; the low number and frequency of patients seen; the preference of less advanced formats of patient materials; and the opinion that there is a lack of educational opportunities for therapists and available resources for patients to access. Further research should delve into these topics and investigate implementation outcomes to ultimately ensure the optimal pelvic health of the population.

## Data Availability

The data generated and analyzed during the current study are available upon request from the author.
